# Respiratory Tract Infections in Diabetes – Lessons From Tuberculosis and Influenza to Guide Understanding of COVID-19 Severity

**DOI:** 10.3389/fendo.2022.919223

**Published:** 2022-07-26

**Authors:** Amnah Al-Sayyar, Katina D. Hulme, Ronan Thibaut, Jagadeesh Bayry, Frederick J. Sheedy, Kirsty R. Short, Fawaz Alzaid

**Affiliations:** ^1^ Dasman Diabetes Institute, Dasman, Kuwait; ^2^ School of Chemistry and Molecular Biosciences, The University of Queensland, St Lucia, QLD, Australia; ^3^ Institut Necker Enfants Malades (INEM), Institut National de la Santé et de la Recherche Médicale (INSERM) U1151/CNRS UMRS8253, Immunity and Metabolism of Diabetes (IMMEDIAB), Université de Paris Cité, Paris, France; ^4^ Department of Biological Sciences & Engineering, Indian Institute of Technology Palakkad, Palakkad, India; ^5^ School of Biochemistry and Immunology, Trinity College, Dublin, Ireland; ^6^ Australian Infectious Diseases Research Centre, The University of Queensland, St Lucia, QLD, Australia

**Keywords:** diabetes, infection, influenza, COVID – 19, tuberculosis, inflammation

## Abstract

Patients with type-2 diabetes (T2D) are more likely to develop severe respiratory tract infections. Such susceptibility has gained increasing attention since the global spread of Coronavirus Disease 2019 (COVID-19) in early 2020. The earliest reports marked T2D as an important risk-factor for severe forms of disease and mortality across all adult age groups. Several mechanisms have been proposed for this increased susceptibility, including pre-existing immune dysfunction, a lack of metabolic flexibility due to insulin resistance, inadequate dietary quality or adverse interactions with antidiabetic treatments or common comorbidities. Some mechanisms that predispose patients with T2D to severe COVID-19 may indeed be shared with other previously characterized respiratory tract infections. Accordingly, in this review, we give an overview of response to Influenza A virus and to *Mycobacterium tuberculosis* (Mtb) infections. Similar risk factors and mechanisms are discussed between the two conditions and in the case of COVID-19. Lastly, we address emerging approaches to address research needs in infection and metabolic disease, and perspectives with regards to deployment or repositioning of metabolically active therapeutics.

## 1 Introduction: Type-2 Diabetes and Severity of Respiratory Tract Infections

Diabetes is a chronic condition characterized by persistently high levels of glucose in blood due to insufficient insulin action or secretion. The international diabetes federation (IDF) estimates that there will be 783 million adults with diabetes in 2045, which is 46% higher compared to the number of cases in 2021 (1). The IDF also reported Type-2 Diabetes (T2D) to be the most prevalent form, accounting for 90% of all cases worldwide (1). T2D is a condition of insulin resistance, defective insulin secretion and β-cell destruction, these are associated with inflammation and metabolic stress. It results from several risk-factors, such as aging, lifestyle and genetic predisposition (2). Metabolic stress due to loss of glycemic homeostasis results in detrimental microvascular and macrovascular complications and hepatic comorbidities (3). Studies have also shown that T2D increases patient susceptibility to severe infections, with this being attributed to microenvironmental dysmetabolism impairing the immune responses (4, 5). Importantly, this applies to several forms of infection: skin and soft tissue infections, urinary tract infections and respiratory infections, with increased hospitalization and mortality rates (6, 7).

Respiratory infections are considered one of the major severe infections associated with diabetes. Hyperglycemia and increased protein glycosylation were found to be associated with microangiopathic alterations in the lungs of patients with T2D (8). Based on the pneumonia severity index, a higher proportion of diabetic patients suffer severe respiratory infections, compared to non-diabetic patients (52.3% vs. 9.4% of patients) (9). In addition, continuous exposure to high glucose also leads to formation of advanced glycation end-products (AGEs) which are implicated in the development of diabetic vascular complications, induce reactive oxygen species (ROS) and development of pulmonary fibrosis (10). These effects in the lung have been observed in common respiratory diseases that gravely affect patients with diabetes. These include asthma (11), pneumonia (7), tuberculosis (12), influenza (5) and Coronavirus Disease (COVID)-19 (13).

In such conditions, immune responses are often studied for potential impairments and as mechanistic targets contributing to severity (14). In normal physiology, immunity coordinates detection of pathogens, phagocytosis, antigen presentation and production of specific antibodies. In diabetes, the increased susceptibility to severe infection has been associated with defects in these functions at multiple levels (4). Results from a cross-sectional study showed that pulmonary functions were reduced in people with inadequate glucose control and high levels of inflammatory markers like TNFα, IL-6 and C-Reactive Protein (15). These inflammatory markers are elevated in diabetes, under non-infectious conditions, and associated with hyperglycemia (16). It was also found that hyperglycemia suppressed IL-2, IL-6 and IL-10 in peripheral blood mononuclear cells (PBMCs) suggesting impaired cellular defense mechanism in patients with diabetes (17). Such markers in circulation reflect localized problems at a given site of infection, such as the lungs. Alveolar macrophages, that populate the lungs, are among the first points of contact with respiratory pathogens, and their dysfunction is a characteristic abnormality in severe cases of COVID-19 (18). These macrophages are specialized tissue resident cells of the innate immune system, generally detecting and disposing of invading foreign debris or pathogens. They also retain the capacity to raise an alarm *via* signaling to cells in the microenvironment (e.g., endothelial or dendritic cells, T-cells etc.).

Every stage of the immune response also relies on micronutrient balance or availability. Micronutrients have synergistic roles with the molecular machinery that facilitates immune effector functions. Vitamin D has been gaining attention as one of the most important micronutrients contributing to efficiency of immune reponses (19). Vitamin D is most widely known as a regulator of calcium and phosphate levels in the body and is necessary for healthy teeth, bones and muscles. However, Vitamin D also ensures integrity of innate immune responses at mucosal barriers, these are the first line of defense against invading pathogens (19). It also plays a role in cell-mediated processes, decrease pro-inflammatory cytokines expression and has inhibitory actions against pathogens (19). The inhibitory effect of the active form of vitamin D [Calcitriol (1,25 (OH)_2_D_3_)] is modulated by vitamin D receptors (VDRs) that are present in the heart, brain, pancreatic islets, immune cells, muscles and adipose tissue. These are all common target organs in the development of diabetes and its complications (20). Typically, during infection immune cells would trigger VDR signaling, to convert vitamin D to calcitriol, then induce the production of antimicrobial proteins such as cathelicidin (LL-37) *via* TLR activation (21).

Several studies have reported an inverse relationship between vitamin D status and T2D incidence (22–24) as its deficiency is associated with β-cell dysfunction and insulin resistance (25). Results from an observational study showed that the prevalence of hypovitaminosis D was higher in T2D compared to control (39% vs 25%) (26). Similarly, Plataki et al. also reported that T2D patients tend to have low levels of vitamin D, as well as of LL-37, providing a mechanism for impaired immune responses and antimicrobial peptide production (27). Such alterations of vitamin D could also increase susceptibility to severe respiratory infections especially in patients with asthma and pulmonary diseases (28). Interestingly, the lung’s epithelium was found to “self-generate” the active form of vitamin D that increases LL-37 expression. Dysfunction of this process can also contribute to severity of respiratory infections (29).

Such immune-mediated mechanisms, and others, have been a central focus of COVID-19 research. Indeed, T2D is one of the top 3 risk-factors leading to severe infection and undesirable outcome of COVID-19 across all adult age groups (13). Interactions between infection, immunity, and metabolic disease has been studied to decipher which criteria contribute to risk and by which mechanisms does diabetes impair response to infection. However, prior to the emergence of SARS-CoV-2, decades of research into respiratory infection in T2D have given a wealth of information. This review brings together current knowledge in T2D and respiratory infections, mainly interactions between systemic metabolism and the immune system’s response to bacterial and viral infectious pathogens. We discuss tuberculosis (TB) and influenza, that predate COVID-19, as longer-studied conditions well-known to be affected by diabetic status. We discuss risk-factors and mechanisms that contribute to severity in both conditions, then relate this to the current knowledge on COVID-19.

## 2 Mycobacterium Tuberculosis and Metabolic Disease

**Figure 1:**
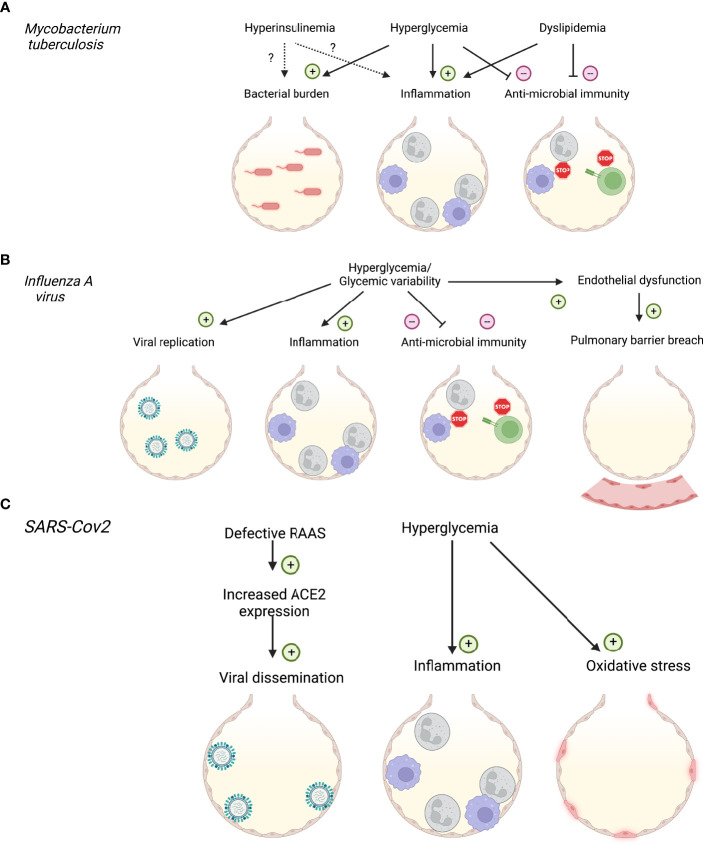
Impact of diabetes and systemic metabolic factors on respiratory infections. **(A)** Impact of hyperinsulinemia, hyperglycemia and dyslipidemia on *Mycobacterium tuberculosis* infection. **(B)** Impact of hyperglycemia on *Influenza A* virus infection. **(C)** Impact of hyperglycemia and renin-angiotensin-aldosterone system (RAAS) dysfunction on SARS-CoV2 infection. Created with BioRender.com.

**Figure 2:**
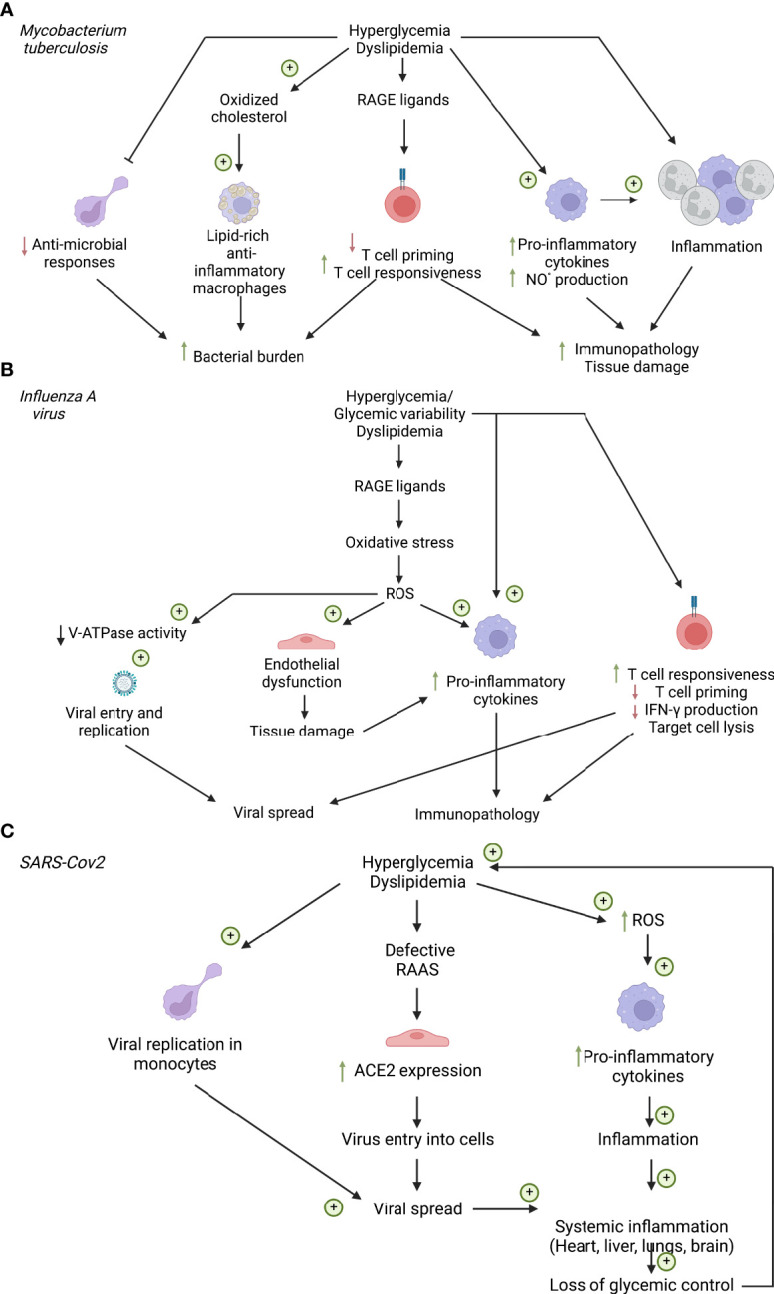
Cellular factors influencing respiratory infections in diabetes. **(A)** Cellular factors influencing *Mycobacterium tuberculosis* infection. **(B)** Cellular factors influencing *influenza A* virus Infection. **(C)** Cellular factors influencing SARS-CoV2 infection. ACE2, Angiotensin converting enzyme 2; NO°, nitric oxide; RAAS, renin-angiotensin-aldosterone system; RAGE, Receptor of advanced glycation end products; ROS, reactive oxygen species. Created with BioRender.com.

The observation that metabolic factors determined the various outcomes of COVID-19, with severe COVID-19 manifesting primarily in people with metabolic syndrome (30, 31), indicates how host metabolic fitness underlines immune function. A similar spectrum of infection outcomes is observed in Tuberculosis (TB), caused by infection with the intracellular bacteria Mycobacterium tuberculosis (Mtb). These range from early clearance to latent tuberculosis infections (LTBI) to active TB and severe disease (32). In recent years, T2D emerged as a major risk-factor for developing severe disease, with TB-diabetes recently classified as a syndrome by WHO, particularly prevalent in Southeast Asia (33). Although both pre-diabetes and T2D can enhance risk of contracting TB in the form of LTBI in low burden TB countries (34), in areas where TB is endemic T2D certainly increases risk of active TB and is associated with poorer responses to treatment (12, 35, 36). Interestingly, in these environments, particularly Asia, T2D is associated with worse TB disease (37) irrespective of body mass index (BMI), while obesity in the absence of hyperglycemia is associated with a better prognosis (38). These observations suggest that multiple factors associated with metabolic syndrome including hyperglycemia, insulin resistance and hyperlipidemia can affect TB host immunity differently and ultimately affect disease outcome. Therefore, TB can serve as a case study for how metabolic factors promote severe infection risk. Insights into metabolic factor contribution to TB immunity, from clinical data and limited animal models, are discussed below (**Figures 1A**, **2A**).

### 2.1 Factors Affecting Tuberculosis in Diabetes

#### 2.1.1 Hyperglycemia

Hyperglycemia affects immune responses to Mtb infection in mouse models and glycemic control has emerged as critical to disease outcome in patient cohorts (**Figure 1A**). Poorer glycemic control is associated with more severe TB disease (39), and strategies to treat this reduce the relative risk. However, glycemia centered strategies do not return relative risk of severe disease to non-T2D levels (40). Mechanistically, peritoneal macrophages from diabetic (db/db) mice display impaired phagocytosis and cytokine responses (41, 42). *In-vitro* studies suggest that increasing glucose concentrations enhance Mtb-induced pro-inflammatory cytokine responses in bone-marrow derived macrophages (BMDM), but this is only noted at high glucose concentrations which mimic hyperglycemia (43) (**Figure 2A**).

With the realization that immunometabolic reprogramming underlines macrophage responses in Mtb, these processes should be re-examined. In particular, monocyte-derived macrophages recruited to sites of Mtb infection up-regulate expression of the main macrophage glucose transporter GLUT1 and glucose consumption to promote anti-microbial functions and pro-inflammatory cytokine production (44, 45). Whether this is impacted by the systemic hyperglycemia, characteristic of T2D has not yet been described. However, circulating monocytes derived from pre-diabetic patients display enhanced cytokine production (46), while monocyte-derived macrophages from patients with T2D have impaired anti-microbial responses to Mtb (47). Similar impairments have been observed in alveolar macrophages derived from diabetic mice (48, 49), these cells seem to rely on oxidative metabolism (45) and are described further in the following sections. *In-vivo* Mtb infections in diabetic mouse models display heightened lung inflammation and bacillary burden (50, 51). This correlates with clinical observations of increased lesions, cavities and increased transmissibility of TB in T2D patients (52, 53). Although the antigen-presenting ability of innate cells was not affected by hyperglycemia (54), altered cytokine production can affect the nature of the subsequent T-cell response contributing to poorer bacterial containment (48). Importantly, *in-vivo* studies have also found a profound defect in inherent lymphocyte function in streptozotocin-treated hyperglycemic mice. Martinez et al. found that the accumulation of Receptor for Advanced Glycation End-Products (RAGE)-ligands during chronic hyperglycemia activates a p38 dependent pathway that epigenetically reprograms naïve T-cells, making them hyper-responsive to subsequent activation by Mtb antigens (54) (**Figure 2A**). Thus, both myeloid and lymphocyte function is inherently impaired under hyperglycemic conditions and can escape the regulatory mechanisms active under homeostasis.

#### 2.1.2 Hyperinsulinemia

Hyperinsulinemia is the result of insulin resistance, given that pre-diabetes is associated with increased risk of LTBI and that cells from pre-diabetic patients display altered responses to stimulation (40, 46), there is an interest in identifying if hyperinsulinemia in pre-diabetes contributes to altered immune responses (**Figure 1A**). The recent interest in immunometabolism has demonstrated that Mtb-infected macrophages upregulate glycolysis to promote anti-bacterial and pro-inflammatory cytokine functions (44, 45), processes enhanced by T-cell-derived IFN-γ and limited by virulent and drug-resistant forms of Mtb (55–57). Although GLUT1 expression is insulin-independent, and insulin-dependent GLUT4 is not expressed in macrophages (58), insulin receptor signaling can have both pro- and anti-inflammatory roles and could impact Mtb immunity. Mtb infection itself can induce transient hyperglycemia through stimulating insulin production which, in guinea pig models, has been linked to more severe disease (59). Identifying these insulin-dependent mechanisms could help explain the complex interdependency between insulin signaling and glucose homeostasis in increased disease severity linked to T2D.

#### 2.1.3 Dyslipidemia

Since glycemic control does not completely reduce risk of severe TB in T2D (40), other metabolic factors have been considered, chiefly, altered lipid homeostasis. Increased BMI contributes to resilience against severe disease (38) and limited human data suggests cholesterol-rich diets may actually protect against TB disease (60). Larger epidemiological studies in Asia have demonstrated that lower total cholesterol levels are linked to more severe TB and poorer treatment outcomes (61). These data suggest a protective role for total cholesterol against TB, which have not been always observed in animal models or *in-vitro* studies. Indeed, hyperlipidemic mice display enhanced TB disease with increased lung inflammation characterized by myeloid nitrous oxide production, increased bronchoalveolar fluid (BALF) Th1 cytokines, poorer lung histology and higher bacterial burdens and dissemination to organs (62, 63)(**Figure 1A**). The increased myeloid inflammation – both macrophage and neutrophil driven, promotes tissue degradation while simultaneously limiting the development of protective T-cell responses. As a caveat, the mouse models used to illustrate this (Apoe-/-, Ldlr-/-; high-fat fed genetically-deficient mice) display abnormally high cholesterol levels, only seen in humans with familial hypercholesterolemia and may not accurately reflect TB-diabetes.

Mtb itself is a lipid rich bacillus and must obtain cholesterol for biosynthesis and growth from the host (64). This promotes long-term persistence in the host since mutant strains which lack cholesterol transporter systems (e.g. mce4) succumb to the pro-inflammatory effects of IFN-γ (65). The TB granuloma, contains a lipid-rich core surrounded by various foamy macrophage cell types (66). Although initially thought to represent a nutrient source for the mycobacteria, recent lipidomics and single-cell analysis suggests that heterogeneity exists in both the lipid content and inflammatory phenotype of granuloma macrophages (67, 68). While Mtb infection can alter both cholesterol and fatty acid metabolism to favor mycobacterial growth (69, 70), the process of lipid droplet formation appears to be host encoded and augmented by IFN-γ treatment (**Figure 2A**). This drives initiation of triglyceride biosynthesis and lipid uptake to maintain lipid droplets in infected macrophages. This process is linked to the production of eicosanoids which promote host-defence (67). Much of this work has been performed in BMDM cultures and it is now appreciated that the alveolar macrophages, initial host cells rely basally on fatty acid oxidation and do not employ anti-Mtb glycolysis, which is the opposite to infiltrating macrophages. Dodd et al. illustrated that Mtb-infected alveolar macrophages and monocyte-derived macrophages upregulate the lipid transporter CD36 to drive an anti-inflammatory, pro-mycobacterial phenotype (71, 72). Surfactant lipoproteins in the pulmonary space facilitate this and are internalized during infection (71). The lipid-rich environment of the lung itself undoubtedly shapes alveolar macrophage development and may explain the tropism of Mtb for the pulmonary compartment. Whether these processes are altered in the setting of hyperlipidemia is unclear and may depend on the nature of the dyslipidemia. Hypertriglyceridemia in western populations was found to be particularly pathogenic and linked to poorer treatment outcomes than hypercholesterolemia (73). Another confounding factor is that often it is not simply elevated lipid levels themselves, but modified lipid species generated during disease (74). Oxidized forms of LDL-cholesterol and sterols have been shown to impact macrophage Mtb responses and macrophage recruitment to the lung respectively (75, 76).

#### 2.1.4 Vitamin D Deficiency

Alterations in vitamin D levels in individuals with diabetes and Mtb can disrupt the maintenance of immune homeostasis. Several studies have reported that cases of Mtb infection are associated with low levels of vitamin D, in particular active Mtb (77, 78). A randomized controlled trial confirmed that upon vitamin D supplementation, patients had significantly reduced symptoms by 36.6% in the first month (79). Since TLRs are linked to the antimicrobial function of vitamin D, Liu *et al.* investigated this mechanism and found that the induction of antimicrobial peptide LL-37 inhibits Mtb proliferation in human monocytes and macrophages (80). This was confirmed further *in vitro* where the inhibition of LL-37 led to enhanced growth of Mtb, confirming LL-37’s suppressive role (81). Very limited number of studies have investigated the status of vitamin D and LL-37 in patients with TB and diabetes. Zhan and Jiang assessed the levels of vitamin D in patients with Mtb and diabetes mellitus and found that the concentration of vitamin D was 35% lower in patients with Mtb and DM compared to the control group (82). In addition, patients with both conditions exhibited high levels of LL-37 that is correlated with a positive acid-fast bacillus smear which indicates the presence of TB (82).

### 2.2 Chronic Inflammation and Immune Training

As a consequence of both hyperlipidemia and hyperglycemia, systemic inflammation is triggered which underlines many of the metabolic sequelae of T2D. This baseline meta-inflammation could also impact host-defense responses and is already implicated in defective anti-tumor immunity (83). Chronic inflammation inhibits both resolution of inflammation and the development of protective lymphocyte responses and can also alter TB granuloma composition (51, 62). Recently, chronic inflammation associated with hyperlipidemia and western diets was shown to reprogram myeloid progenitor cells for heightened activity through the process of innate immune training (84, 85). Following this, Choudhury and colleagues demonstrated that hyperglycemic but not hyperlipidemic mice also displayed heightened monocyte cytokine production due to epigenetic modification and alterations to bone-marrow myelopoiesis, characteristic of innate immune training (84, 86). This process contributes to the development of T2D, but may also impair host-defense in TB-diabetes. With hematopoietic progenitor cells emerging as a key target of inflammation affecting immune cell composition and fate (87), the chronic inflammation in T2D could alter immune cell fate and affect granuloma composition. Recent profiling studies examining active TB granulomas demonstrate a more regulatory phenotype dominated by immunosuppressive myeloid and Treg cells (68) and the impact of meta-inflammation on this warrants examination.

### 2.3 (Immuno)-Metabolic Treatments for TB-Diabetes

Although control of glycemia in T2D doesn’t completely reduce the risk of increased TB (40), metabolic drugs are being assessed for their ability to prevent disease due to their immunometabolic effects. They can also reveal novel targets in pathogenesis which could form the basis for future novel immuno- therapies or vaccine targets. In a normoglycemic mouse model of TB, metformin was shown to exacerbate late-stage disease by ameliorating inflammation (88). In particular, IL-1β expression was reduced, which can be regulated through metformin-induced inhibition of mitochondrial complex 1 activity (89). Similar results were observed in humans receiving metformin, with improved macrophage responses to Mtb infection (90). More pressingly, in the context of T2D, a recent meta-study revealed that diabetes controlled by metformin led to a reduced risk of TB in humans (91). In contrast, a recent mouse study of Mtb infection in DIO-mice suggests that metformin is only beneficial in the hyperglycemic context (92). More recently, cytotoxic CD8^+^ lymphocytes emerged as targets for metformin in Mtb-infection models in euglycemic mice. Metformin metabolically reprograms these cells toward oxidative metabolism and promotes anti-microbial function. Traditionally TB vaccines target CD4^+^ Th1 cells, so this study is important as it places these CD8^+^ T-cells as key in protecting against severe disease, particularly relevant in the metabolically dysregulated context of T2D.

Because of Mtb’s tropism for cholesterol, the HMG-CoA reductase targeting drugs, statins, have received attention both as a direct anti-Mtb drug and for use in the context of TB diabetes. By reducing cholesterol biosynthesis at this early rate-limiting step (93), statins lead to compensatory LDLR expression and reduced serum LDL-cholesterol which should be beneficial in the context of hyperlipidemia and its effect on TB pathogenesis. Clinical studies are examining the feasibility of this (94), however given the controversial role of elevated total cholesterol in TB host defense, the results thus far are unclear. Since targeting HMGR leads to knock-on effects on the production of multiple lipid species (93, 95), statins may affect mycobacterial growth. *In vitro* studies suggest that statins may directly alter axenic Mtb growth in culture (96), Mtb growth in macrophages is also reduced after statin treatment (97). This may be due to cholesterol depletion but also could be mediated through the immunomodulatory functions of statins (95). Finally, novel lymphocyte subsets which present lipid antigens *via* CD1 have recently been associated with early immune responses in TB including γδ T and iNKT cells (98). The impact of altered systemic lipid homeostasis as well as statin treatment on their function needs to be considered, as does the nature of vaccine strategies used to boost TB immunity, with current targets focusing solely on MHC-restricted peptide antigens that promote CD4^+^ T-cells.

## 3 Influenza A Virus and Metabolic Disease

Influenza A virus (IAV) presents a continuous threat to human health, infecting up to 15% of the population annually (99, 100). Influenza infection severity largely depends on the immune and health status of the individual (101). Although considered to be relatively mild, the 2009 H1N1 pandemic served to emphasize that certain host comorbidities increase the risk of severe disease upon influenza virus infection (102, 103). Prior to 2009, reports already emphasized that diabetes increases the risk of lower respiratory tract infections (LRTI) and pneumonia related hospitalization, and indeed led to more severe influenza outcomes (104–106). Both patients with T1D and T2D were at greater risk of developing LRTI (including influenza), while there was no difference in the risk of upper respiratory tract infections over a 12-month time period (105). Interestingly, in this study T2D patients in particular had an increased risk of developing two or more episodes of a LRTI within the 12-month period. This indicates that hyperglycemia *per se* may be an important factor in LRTI severity, but not necessarily in frequency of LRTIs. A 7-year retrospective study reported that individuals with diabetes are at a higher risk of being hospitalized due to pneumonia (104). This increased risk was also associated with increasing blood glucose levels (104). Furthermore, during a one year period, individuals with diabetes were more likely to have influenza or pneumonia listed as cause of death compared to people without diabetes, regardless of race, sex, and socioeconomic status (106). Strikingly, it was estimated that during this time, individuals with diabetes made up more than 10% of the recorded influenza and pneumonia associated deaths in the U.S (106). Together, these studies demonstrate the enhanced risk of severe influenza outcomes in individuals with diabetes during seasonal influenza epidemics.

Much of the current literature regarding the susceptibility of individuals with diabetes to severe IAV infection emerged in response to the 2009 H1N1 pandemic (pH1N1) and has been addressed in a previous review (107). Specifically, diabetes was identified as one of the most common host comorbidities in those hospitalized with pH1N1 (108). During the 2009 pandemic, individuals with diabetes had around a 3-4-fold increased risk of hospitalization (109–112), intensive care unit (ICU) admission (109, 113, 114), and death (113–115). Indeed, one study reported up to a 9-fold increased risk of hospital mortality in individuals with diabetes (116). While diabetes was identified as an independent risk-factor for severe pH1N1 outcomes, this risk of ICU admission and death becomes higher in patients with additional underlying medical conditions (117).

### 3.1 Factors Influencing Influenza in Diabetes

#### 3.1.1 Hyperglycemia

Experimentally, murine models of both T1D and T2D show that diabetic mice have more severe influenza outcomes when compared to healthy controls (107, 118–120), providing experimental evidence that hyperglycemia *per se* is a major contributor to disease severity. Studies using an *in vivo* STZ-induced model of T1D, demonstrated that diabetic mice had increased viral load with a more extensive infection, and lower rates of survival (118, 120, 121). This increase in viral load has also been correlated to increasing blood glucose levels observed in T1D mice (119). Similar results have been observed using a leptin receptor-deficient model for obesity and T2D. Specifically, elevated blood glucose levels correlated with increased viral copy number and an increase in the pulmonary pro-inflammatory cytokine response (122) (**Figure 1B**).

Despite the abundance of data that indicates the role of diabetes in influenza severity, the research into mechanisms remains limited. Chronic hyperglycemia can induce oxidative stress *via* the production of ROS and is thought to be one of the key sources of hyperglycemia-induced diabetes complications (123, 124). There are several mechanisms by which hyperglycemia causes increased oxidative stress, such as the production of AGEs, the activation of Protein Kinase-C (PKC), the accumulation of sorbitol, and the hyperactivity of the hexosamine pathway (125). Cumulatively, these lead to the over production of ROS and a decrease in the endogenous antioxidant defense systems (125). Given the causal role of hyperglycemia-induced ROS has in the development of the non-infectious complications of diabetes, it is likely that hyperglycemia-induced ROS production is a driving factor in increased severity of influenza in individuals with diabetes (**Figure 2B**).

There is evidence that hyperglycemia-driven ROS production alters cellular metabolism that can shape the inflammatory response during viral infections (126), however this has yet to be explored in influenza. Hyperglycemia can upregulate glycolysis (127), which can promote a pro-inflammatory immune cell phenotype (128), and increase viral replication (129) (**Figure 2B**). Despite not coming in direct contact with circulating blood, glucose concentrations in the airway surface liquid increase as a direct result of elevated blood glucose levels (130). High levels of glucose cause a dose-specific increase in IAV infection and replication in Madin-Darby Canine Kidney (MDCK) epithelial cells (129). This was associated with a glycolysis-dependent increase in the assembly of the cellular V-ATPase which is necessary for viral release into the cytoplasm (129). Indeed, increased viral replication has been observed in murine models of both T1D and T2D, and correlated with increased blood glucose levels (119–122). This increase in viral replication as a result of abundance of glucose is likely due to ROS-driven alterations to metabolic function. While this is yet to be directly demonstrated in the context of influenza, high levels of glucose have been shown to increase SARS-CoV-2 viral replication and monocyte cytokine production in a ROS/glycolysis-dependent manner (131).

In addition to altering cellular metabolic function, a history of exposure to hyperglycemia can lead to ROS-driven endothelial dysfunction in diabetes (132). While endothelial cells are not normally infected by influenza virus in humans, they play a crucial role in influenza pathogenesis given their close proximity to the pulmonary alveolar epithelium. Specifically, during IAV infection, it is the pulmonary endothelial cells that are believed to be a major source of cytokine production in the lungs (133, 134). We have previously demonstrated using both an *in vitro* and *in vivo* model, that exposure to high glucose conditions prior to IAV infection increased virus-induced pulmonary barrier damage (122). This was associated with an increased pro-inflammatory response in endothelial cells and the subsequent damage of the epithelial junctional complex. This is likely to be further exacerbated in conditions of glycemic variability (**Figure 2B**).

#### 3.1.2 Glycemic Variability

Typically, individuals without diabetes have blood glucose levels ranging between 70-100 mg/dl (135). These glucose levels fluctuate during the day, particularly post-meal up to almost 140 mg/dl (135). These glucose fluctuations are referred to as glycemic instability or glycemic variability and can be more extreme and prolonged in individuals with uncontrolled diabetes.

There is mounting evidence that glycemic variability is associated with greater ROS production than steady hyperglycemia (125), and could further impair the immune response (107, 136). However, glycemic variability as a factor in severe influenza infections remains a relatively new topic, with an extremely limited amount of research into its role, and most studies are outside the scope of influenza. Nevertheless, there has been one key study that has investigated the role of glycemic variability in the context of influenza. Using an *in vitro* co-culture model mimicking the respiratory epithelial-endothelial barrier we have shown that compared to hyperglycemia, glycemic variability increased viral replication, cell death, and inflammation of both the epithelial and endothelial cells (136). This was correlated with an increase in a marker of oxidative stress. These results were confirmed using an *in vivo* model, where mice that experience glycemic variability in the weeks leading up to infection suffered more severe influenza (136). Specifically, mice with glycemic variability had increased weight-loss, decreased lung function, and increased apoptotic cell death. This was again associated with increased pulmonary inflammation and oxidative stress. Together, these data suggest a key role of dysregulated glucose levels, both elevated and variable, driving severe influenza in the context of a primary infection (**Figure 1B**).

### 3.2 Adaptive Immune Responses

In addition to its effect on the innate immune system and viral replication, diabetes may also have direct effects on the cellular adaptive immune response to viral infection. There is evidence that hyperglycemia induces hyperresponsiveness, enhanced activation, and proliferation of T cells (54, 137) (**Figure 2B**). However, there is perhaps a larger body of evidence that hyperglycemia impairs responses, increases the frequency of senescent cells, and impairs the proliferation of T cells (138–140). *In vitro* evidence suggest that high glucose concentrations reduce the production of IFN-γ by CD8^+^ T cells (141), and reduces their viability (142). Consistent with this, it has been shown that when compared to healthy controls, CD8^+^ T cells from patients with diabetes had reduced lysis of target cells (143), and using genome-wide expression analysis of PBMCs from donors with diabetes showed a reduction in activity of cytotoxic genes compared to controls (144). Much like what has been observed in innate immunity, there is an emerging role of glycemic variability in negatively shaping the adaptive immune response to influenza. Compared to hyperglycemia, we have shown that glycemic variability increases pulmonary inflammation, oxidative stress and influenza severity following a secondary influenza infection in a murine model (136). It was speculated that this increase in severity could be driven by glycemic variability-induced oxidative stress reducing the CD8^+^ T cell function. Currently, there is very little known about the effect of both hyperglycemia and glycemic variability on the adaptive immune response to influenza, and further in-depth investigation is warranted.

Taken together these studies suggested that individuals with diabetes suffer more severe influenza as a result of i) metabolic dysfunction, ii) increased viral replication, iii) endothelial dysfunction, and iv) dysregulation of the immune response.

### 3.3 Response to Vaccination in Diabetes

As individuals with diabetes are at a higher risk of developing serious influenza complication, influenza vaccination is currently recommended by the Centers for Disease Control and Prevention (CDC) for patients with diabetes (145). During recent influenza seasons, almost 30% of adults hospitalized with influenza had diabetes (145), highlighting the need for vaccination in this vulnerable group. During influenza epidemics of the early 1990s, vaccination of individuals with diabetes (both T1D and T2D) reduced hospital admission by almost 80% (146). A more recent meta-analysis determined that when individuals with diabetes (both T1D and T2D) are vaccinated, there is a reduced risk of hospitalization for pneumonia, and a lower mortality rate (147). Furthermore, while individuals with diabetes are at risk of developing cardiovascular complications following influenza virus infection (148, 149), influenza vaccination is associated with a reduced risk of cardiovascular mortality in adults with diabetes (150). While there are rare case study reports of adverse reactions to influenza vaccination in individuals with diabetes, overall, reactogenicity is similar in individuals with diabetes and healthy adults (151, 152). Although influenza vaccines are proven to be safe and reduced the development of severe complications, there have been some questions over vaccine efficacy in people with diabetes. Specifically, there is evidence that whilst patients with T2D were more likely to have received the influenza vaccination in the last 12 months they still experienced a greater number of respiratory infections than their non-diabetic counterparts (153). This is likely due to their reduced T cell and antibody response to influenza vaccines. In a small cohort, patients with T1D had decreased T cell response to influenza A-H1N1 subunit vaccine compared to controls, and this was associated with hyperglycemia (143). A larger study that encompassed both T1D and T2D reported that in the T1D group there was a significant increase in antibody non-responders to two of the three vaccine components (143). While this may be cause for concern, it has been demonstrated *in vivo* that vaccination *via* a higher dose, or a second low dose, increases antigen levels in diabetic mice to the point that they are able to protect against an otherwise lethal challenge (120, 121). Together, this emphasizes the importance of maintaining up-to-date vaccination in individuals with diabetes. Unfortunately, vaccination rates for influenza are often lagging amongst those with diabetes. The current target vaccination rate for patients with diabetes is 75% (99). However, in developed countries coverage values are around 50-70% (154, 155), with some as low as 10% (156). Recently, in China, the reported vaccination rate of individuals with diabetes was below 8%, despite more than 46% of participants reporting an original intention to receive the vaccine (157). On top of lagging vaccine coverage rates in individuals with diabetes, the overall efficacy of the vaccine is known to change from season to season, as the circulating strains change. For example, the CDC has reported the current influenza vaccination for the 2021-2022 flu season in the U.S. did not reduce the risk of outpatient illness caused by influenza A (H3N2) (158). This suggests that not all influenza associated complications and death in diabetic individuals can be prevented with vaccination alone. Lagging vaccination rates, combined with sub-optimal vaccine efficacy in some influenza seasons means that severe influenza infections remain an issue for those living with diabetes.

## 4 COVID-19 Severity and Type-2 Diabetes

The above examples of respiratory infections in patients with diabetes clearly indicate a strong interaction between metabolism and successful detection and clearance of invading pathogens from the lung. Whether this be at the level of systemic metabolism or cellular immunometabolic defects that alter immune responses, a wealth of knowledge from cases of influenza and of TB can be used to draw comparisons with COVID-19.

Global events since March 2020 have made COVID-19 a near singular preoccupation of medical and research professionals due to the unprecedented strain on healthcare services. Early in the outbreak, T2D and associated metabolic syndrome were identified as risk factors for severity and death from COVID-19 (159). Diabetes is underpinned by inflammation and systemic dysmetabolism putting patients at-risk of other comorbidities. Although, initial reports focused on the immunology of SARS-CoV-2 infection and clinical trials applied antiviral or anti-inflammatory therapies (160); higher mortality in patients with pre-existing dysmetabolism indicates that metabolic mechanisms are also attractive, to establish risk, or redeploy therapeutics to mitigate severity. Indeed, the increased severity of other respiratory infections (e.g., TB, influenza) in diabetes and the interactions with specific metabolic traits support those common features of metabolic decline that are more important to focus on to mitigate risk.

The earliest report dedicated to COVID-19 patients with T2D revealed that glycemic instability/variability increases risk of severe disease (13). Further meta-analyses found that of the components of metabolic syndrome, diabetes is the biggest contributor to adverse outcome compared to hyperlipidemia, obesity and hypertension (161). Multiple reviews have addressed the interactions between COVID-19 and T2D (162, 163). Here we will cover common points with risk factors for TB and influenza, as well more recent questions regarding metabolically active therapeutics and responses to vaccination in the population with TD2 and COVID-19.

### 4.1 Factors Influencing COVID-19 in Diabetes

Identified factors that increase risk of COVID-19 include increased inflammation, increased ROS, insulin resistance, hyperglycemia and vascular endothelial damage. All of these factors are pre-existent or accentuated in patients with T2D prior to infection with SARS-CoV-2 (163). These risk factors are generally shared with IAV and Mtb infection (**Figures 1C**, **2C**). The particularity of SARS-CoV-2 is its activation of the renin-angiotensin-aldosterone system (RAAS) *via* the virus’s main entry point, the Angiotensin converting enzyme (ACE)-2 (164). The RAAS is defective in T2D and has long been known to contribute to the development of complications in diabetes (nephropathy, macroangiopathy) (165, 166). However, dysfunction of this system cannot solely explain the increased susceptibility of patients with diabetes to a plethora of infectious respiratory conditions. Therefore, other metabolic factors are valuable candidates to look into to better understand risk.

#### 4.1.1 Hyperglycemia

Hyperglycemia increases SARS-CoV-2 replication in circulating monocytes (131). Interestingly, on the immunometabolic front, mitochondrial adaptation also occurs in these cells to produce more ROS, contributing to severity. Our own work demonstrated that patients most severely affected exhibit morphological and functional changes in their monocyte pool (167). These changes are related to hyperinflammation and interferon signaling, associated with severity and are more pronounced in T2D. Thus, potentially *via* an interaction with monocytes, hyperglycemia may be an important mechanism-based risk-factor for severe COVID-19. Of note, glycemic control tends to deteriorate with infection in patients with T2D (**Figure 2C**). Those on insulin will require increasing doses to lower glycemia, and this requirement is associated with increased levels of inflammatory cytokines (168, 169).

Local and systemic inflammation are characteristic of COVID-19. Autopsy studies have reported inflammatory infiltrate, in the form of macrophages and lymphocytes, in a number of tissues, including the lungs, myocardium, liver and the brain (170, 171). Systemically, the cytokine storm syndrome and hyperinflammation are common and potentially life threatening in severe cases (172). The major immune signals that are impaired in COVID-19 at the transcriptional and translational levels are pro-inflammatory IL-6 signaling and the type-1 interferon system (173, 174). Mechanisms shared with other coronavirus infections have been proposed to link immune responses to disturbed metabolic homeostasis and worsening disease course. Notably, the large burden of inflammatory infiltrate affects key insulin-responsive tissues, including the liver and skeletal muscle (175).

#### 4.1.2 Dyslipidemia

The role of dyslipidemia in COVID-19 severity on a background of T2D is unclear. Studies have found either increased risk of severe COVID-19 with dyslipidemia or no effect (176). The lack of clarity comes from confounding factors (such as multiple treatments, age and comorbid conditions) and from heterogeneity of studied populations.

#### 4.1.3 Vitamin D Deficiency

Hypovitaminosis D is associated with severe COVID-19, increased hospitalization and increased mortality (177). Several studies reported the inverse relationship between vitamin D supplementation and COVID-19 and its role as a preventive measure (178, 179). Generally, the protective effects of vitamin D against COVID-19 are linked to respiratory epithelial cell production of LL-37 that contributes to host-defense mechanisms through the disruption of viral membranes and replication (180). This was confirmed by Roth et al. as they demonstrated the role of LL-37 in preventing viral cell entry by binding to the ACE2 receptor of SARS-CoV-2 (181). It was also noted that vitamin D deficiency leads to a reduction in modulatory potential towards the cytokine storm during viral infection. This phenomenon is implicated in braking the lung epithelium, resulting in alveolar edema (182).

To date, very limited data are available on the link between hypovitaminoses in diabetic individuals and COVID-19. Results from a clinical study found that 76% of patients with vitamin D deficiency and hyperglycemia had severe COVID-19 with increased hospitalization and mortality rates compared to patients with normal vitamin D and glucose levels (183). They also confirmed that hypovitaminosis D in hyperglycemia resulted in worse respiratory parameters and increased levels of IL-1b, IL-6 and IFN-γ (183). Similar results were also found in obese and vitamin D deficient individuals with 72% increase in infection severity compared to the control group (183). These studies suggest that optimal nutrition and supplementation with vitamin D is a promising candidate as a preventive measure or potentially an adjunct treatment for COVID-19 (184).

#### 4.1.4 Obesity

Obesity is considered as a major risk factor for COVID-19 infection due to its significant role in increasing systematic inflammation through the dysregulation of adipose tissue. Compared to non-obese individuals, obese individuals had greater COVID-19 severity, hypoxemic respiratory failure and higher baseline initial serum levels of C-reactive protein and IL-6 (associated with low-grade-chronic inflammation) (185). This suggests that obesity leads to worse COVID-19 outcomes that are associated increased inflammation and metabolic dysregulation.

### 4.2 Response to Vaccination and Metabolically Active Therapeutics

Efficiency of the COVID-19 vaccine response has been evaluated in patients with T2D. Vaccination has been reported to be efficient in patients with good glycemic control compared to patients with uncontrolled T2D. Therefore, the adaptive immune compartment does not seem to be compromised with regards to antibody production in T2D. However, hyperglycemia in uncontrolled diabetes results in weak immunity, indicating that poor glycemic control can indeed impair the antibody producing branch of adaptive immunity. Two independent studies reported similar results (186, 187).

Of the different classes of metabolically active drugs applied in metabolic syndrome and T2D, some have been studied for their contribution to risk or better outcome of infection with SARS-CoV-2. Given the risk factors described above, one would hypothesize that rigorous glycemic control and use of insulin would be beneficial, whilst use of lipid lowering drugs may not affect outcome. Metanalyses have found that user of metformin and sulfonylureas had lower mortality risk (188). Metformin acts by lowering hepatic glucose output, with a number of proposed molecular mechanisms, and Sulfonylureas are insulin secretagogues. Interestingly, some studies report that insulin use was associated with worse outcome (188). This is at odds with the proposed action on stimulating CD8^+^ T-cell function, however in an already diabetic context, the use of insulin may be a sign of progressed diabetes and thus an overall less healthy metabolic status of the patient. Use of dipeptidyl peptidase-4 inhibitors did not have an effect on disease outcome. So, these studies indicate that use of antidiabetic agents either improve or do not affect outcome in COVID-19 in patients with T2D; unless patients with T2D have progressed to the stage of use of exogenous insulin. With regards to controlling dyslipidemia, the most commonly used class of drugs is Statins and studies have been ambivalent. Some meta-analyses conclude better outcome from retrospective studies (189) whilst others report improvement, worsening or no effect (190).

## 5 Perspectives: Metabolomic Shifts and Metabolic Depression in COVID-19

Beyond pre-existing T2D, and clinical and immunological factors mentioned above, another way of addressing interactions between metabolism and response to infection is the application of metabolome studies. Such approaches are most valuable to basic research when applied translationally, studies are often data-driven in nature and when combined with clinical and immunometabolic observations, metabolomic approaches may lead to further hypothesis generation to direct future mechanistic work. Two reports by Shen et al (191) and by Wu et al (192) actually characterized the systemic metabolomic, lipidomic and proteomic responses to SARS-CoV-2 infection. Concertedly, these studies show that COVID-19 is characterized by a generalized systemic metabolic depression, although they were carried out in non-diabetic patients, metabolomic and lipidomic changes may indicate which specific pathways are dysregulated in COVID-19, and potentially subject to worsening in patients with metabolic disease.

The study by Shen et al. investigated proteomic and metabolomic profiles of sera from patients with severe and non-severe COVID-19 and non-COVID-19 patients with similar clinical presentation (other respiratory infections). In this study, the COVID-19-dependent proteome and metabolome represented three major pathways: complement system, macrophage function and platelet degranulation (191). Lipoproteins, sphingolipids, glycerolipids, steroid hormones and their intermediates related to macrophage function were downregulated in COVID-19 and correlated to severity. These molecules play important roles in immune function, including signaling, regulating membrane properties, apoptosis, migration and importantly in the resolution of inflammation. This lipid repressive profile is specific to SARS-CoV-2, as studies with other viruses (HCoV-229E, MERS-CoV) found increases of several of these lipid mediators relative to healthy subjects (193). As for liver-derived molecules, increased bilirubin degradation products and bile acid derivatives indicate impaired hepatic detoxification and urea cycle activity is also altered, which is a typical consequence of the interferon response associated with viral infection. Interestingly, the proteomic profile also showed decreased serotonin correlating to disease severity; which would influence sickness behaviors (e.g., lack of appetite, lethargy) also altering systemic metabolism.

The publication by Wu et al (192) carried out kinetic targeted lipidomic and metabolomic profiling of sera from healthy subjects and from COVID-19 patients ranging from mild to severe, and to fatal disease. When compared to healthy subjects, 87 out of 431 metabolites tested were altered in patients with fatal COVID-19 at inclusion, which increased to 162 at last sampling; with fewer metabolites altered in severe and mild cases. Pathway analyses over all severities reflected enrichment of pyrimidine, fructose and mannose and carbon metabolism as well as taste transduction pathways; whereas fatal case features reflected thyroid hormone synthesis and signaling, and purine metabolism pathways. The last time points sampled in fatal cases were marked by an acute reduction of metabolites, with malate and aspartate being the most affected, indicating breakdown of mitochondrial respiration. Targeted lipidomic analyses found most lipids to be upregulated in COVID-19 compared to healthy subjects, with magnitude increasing with severity. Lipids dysregulated over all severities enriched pathways in phosphatidylinositol signaling, inositol phosphate metabolism and long-term depression. Dysregulated lipids in fatal COVID-19 enrich endocannabinoid signaling, bacterial and viral infection and glycerophospholipid metabolism pathways. Interestingly, the serum metabolome did not normalize after recovery. This could be due to residual effects of hyperinflammation, lasting damage to metabolic tissues, a particularity of COVID-19; or a combination of the above. In any case, this indicates that clinical recovery is not dependent on re-establishing metabolic homeostasis.

Taken together the above studies report generalized metabolic depression in COVID-19. Although metabolic diseases were not discussed, it is reasonable to assume that pre-existing dysmetabolism, sub-optimal liver function, impaired glucose homeostasis, dyslipidemia, or any other disequilibrium associated with metabolic disease will adversely affect the metabolic response to COVID-19. The above studies indicate that higher risk of severity in metabolic disease may be due to a lack of metabolic flexibility, as required to efficiently respond to SARS-CoV-2 infection. Indeed, the 80% of COVID-19 cases that are mild or asymptomatic, may be so because of their adaptable metabolism, capable of ramping up or down their substrate production or utilization to cope with pathogen burden.

Several overarching themes can be drawn in the context of these studies. COVID-19 is associated with: 1) Compromised liver function; 2) Dyslipidemia; 3) Depression or a depression-like molecular profile; and 4) Altered cellular metabolism. The liver response in releasing alarm molecules is common in severe infection and sepsis, compromised function may arise from high viral loads, congestion due to outstripped filtering capacity, or pre-existing intrahepatic triglycerides that impair liver function. Dyslipidemia may be due to increased lipolysis from a highly inflammatory background, which would be aggravated in T2D where fatty tissues lose sensitivity to insulin’s anti-lipolytic effects. Importantly, dyslipidemia and increased lipolysis may also arise due to sickness behaviors that modify energy intake and expenditure, notably a loss of appetite and drop in blood glucose levels causing ketogenesis and reliance on lipid metabolism. Finally, effects were reported on cellular pathways of nucleic acid synthesis, lipid synthesis and oxidative metabolism. The loss of substrates for nucleic acid and lipid synthesis, may be explained by virus-host cell dynamics where viral particles usurp nucleotides for their own replication, and make use of cellular lipids to construct their envelopes. SARS-CoV-2 seems particularly efficient in diverting the use of cellular substrates for its own needs, the authors describe this as a ‘hijacking’ of host cell metabolic machinery (191). A recent study by Zhang et al. supports such dynamics by demonstrating that intercellular glucose and folate are depleted in SARS-CoV-2 infected cells. This is the result of SARS-CoV-2 infection orienting glucose and folate metabolism towards needs supporting viral replication, destabilizing host mRNA abundance and protein translation processes by its use for viral biosynthesis (194).

To put findings into context, over recent years immunometabolism research has characterized systemic immunity in metabolic diseases as well as cellular metabolism associated with immune functions. Yet the metabolic demands of viral infection remain relatively understudied. A hallmark publication by Wang et al. in 2017 (130) highlights the systemic metabolism associated with bacterial and viral infection. Whilst both cause similar symptoms or sickness behaviors: loss of appetite, weight loss, fever, lethargy; the energy imbalance and systemic metabolic shift is favourable only in bacterial infection. Of note, fever and marked weight loss are common to SARS-CoV-2, where decreased energy intake is aggravated due to the loss of taste and smell and depression of central reward systems with decreased serotonin reported in the above studies (191, 192, 195). The resulting undernutrition shifts systemic metabolism from glucose-dependence to reliance on ketone bodies and fatty acids, reminiscent of fasting metabolism. This is maladaptive in viral infection, where nutritional supplementation, particularly with glucose, is protective, independently of inflammatory status or pathogen burden. The systemic metabolic shift in infection is also favorable for viral replication, where free fatty acid substrates are used to form and close virion membranes (196). Whilst all may also apply to COVID-19, the added respiratory depression will also starve the host of oxygen and thus the capacity to maintain efficient oxidative metabolism, largely dependent on lipid-substrates, the mediators of which also sustain lipogenesis.

To date, few specific therapies have proven to be effective against COVID-19 (197, 198). Generally patients receive standard supportive care and antiviral therapies, trials have been ambivalent with regards to alternative therapies including chloroquine, steroids or their related compounds (199). These studies and many others have applied these approaches to classify patient risk, importantly these findings can also be used to redirect trials towards metabolically active therapeutics already applied in metabolic diseases. Analyses from medical databases based around prescription history will also give signs whether lipid lowering, glycogenolytic or anti-hyperglycemic agents may mitigate or aggravate COVID-19, as well as other respiratory infections with a high burden of disease.

## Author Contributions

AA, FA, RT, FS, KH, KS, JB wrote the manuscript. All authors contributed to the article and approved the submitted version.

## Funding

This work was supported by the Kuwait Foundation for the Advancement of Sciences (KFAS) grant (RA HM-2019-009). FA was supported by the French National Research Agency (Agence Nationale de la Recherche; ANR) ANR-JCJC grant for the MitoFLAME Project ANR-19-CE14-0005. RT was supported by a grant from the European Foundation for the Study of Diabetes (EFSD). FS was supported by Science Foundation Ireland Award 19/FFP/6625. KS is funded by NHMRC investigator grant 2007919.

## Conflict of Interest

KS is a consultant for Sanofi, Roche and NovoNordisk.

The remaining authors declare that the research was conducted in the absence of any commercial or financial relationships that could be construed as a potential conflict of interest.

## Publisher’s Note

All claims expressed in this article are solely those of the authors and do not necessarily represent those of their affiliated organizations, or those of the publisher, the editors and the reviewers. Any product that may be evaluated in this article, or claim that may be made by its manufacturer, is not guaranteed or endorsed by the publisher.
